# Postoperative watershed shift induced ischemic stroke after direct revascularization surgery in chronic intracranial atherosclerotic steno-occlusive diseases; case series and literature review

**DOI:** 10.1007/s00701-025-06715-0

**Published:** 2025-12-02

**Authors:** Masahiko Nishitani, Taichi Ishiguro, Shunsuke Nomura, Yoshihiro Omura, Kostadin Karagiozov, Tadasuke Tominaga, Nobuhiko Momozaki, Mana Suzuki, Akitsugu Kawashima, Takakazu Kawamata

**Affiliations:** 1 Department of Neurosurgery, Tokyo Women’s Medical University Yachiyo Medical Center, Owadashinden 477-96, Yachiyo-Shi, Chiba, 2768524 Japan; 2 Krembil Research Institute, Universtiy of Toronto, Toronto, ON Canada; 3 Department of Neurosurgery, St. Luke’s International Hospital, Tokyo, Japan; 4 Department of Neurosurgery, Tokyo Women’s Medical University, Tokyo, Japan

**Keywords:** Atherosclerosis, Bypass, Infarction, Middle cerebral artery, Superficial temporal artery

## Abstract

**Background:**

Extracranial-intracranial (EC-IC) bypass surgery is performed to reduce the risk of ipsilateral cerebral infarction in selected patients with chronic intracranial atherosclerotic steno-occlusive disease (ICAD) with reduced cerebral blood flow (CBF). However, postoperative watershed shift induced ischemic stroke (WSIS) may occasionally occur despite maintained bypass patency and improved CBF. We report the incidence and characteristic features of WSIS after superficial temporal artery-middle cerebral artery (STA-MCA) bypass for chronic WSIS.

**Methods:**

We retrospectively analyzed 158 patients with symptomatic chronic ICAD and impaired CBF and cerebrovascular reactivity who underwent STA-MCA bypass between 2013 and 2023. Clinical data and pre- and postoperative imaging findings were analyzed to identify WSIS.

**Results:**

Postoperative bypass patency was 100%. Ischemic complications occurred in 3 of 158 patients (1.9%), all of which were WSIS. Notably, 3 WSIS cases occurred in patients with severe internal carotid artery stenosis. These infarctions occurred on postoperative day 3, despite good bypass patency. Angiography confirmed bypass flow supplied the entire MCA, but anterograde ICA flow was consequently reduced, leading to a hemodynamic shift.

**Conclusions:**

WSIS is a rare (1.9%), but important complication after STA-MCA bypass, occurring in patients who have preserved anterograde flow preoperatively.

## Introduction

Extracranial-intracranial (EC-IC) bypass surgery for intracranial atherosclerotic steno-occlusive disease (ICAD) remains controversial [11, 16]. In our country, it is performed selectively in chronic ICAD patients who exhibit decreased cerebral blood flow (CBF) and impaired cerebrovascular reactivity (CVR), as EC-IC bypass has been shown to reduce the risk of ipsilateral cerebral infarction in this population [15]. Following successful bypass, the donor artery provides robust collateral blood flow to the anterior circulation, thereby improving CBF. However, in rare instances, ipsilateral cerebral infarction may still occur postoperatively despite maintained bypass patency and increased cortical perfusion. Hemodynamic alterations have been proposed as a likely mechanism underlying this type of cerebral infarction. In particular, retrograde blood flow from the bypass may reduce anterograde perfusion by supplying a broad area of the anterior circulation, resulting in displacement of the “watershed” area. This article presents illustrative cases of such paradoxical infarction—termed watershed shift induced ischemic stroke (WSIS)—following EC-IC bypass in patients with ICAD. It also explores the characteristics of this phenomenon through a comprehensive review of the literature.

## Materials and methods

We retrospectively analyzed 194 patients with ICAD who underwent extracranial-intracranial (EC-IC) bypass at our institution between April 2013 and March 2023. This study included only patients who underwent surgery during the chronic phase; 36 patients who underwent surgery in the acute phase of cerebral infarction were excluded.

Surgical indications for EC-IC bypass were as follows: 1. Age ≥ 18 years, 2. Preoperative modified Rankin Scale (mRS) score of 0–2, 3. Severe stenosis or occlusion of the internal carotid artery (ICA) or M1 segment of the middle cerebral artery (MCA), 4. Symptomatic ischemic stroke or transient ischemic attacks, 5. Decreased resting cerebral blood flow (CBF) and impaired cerebrovascular reactivity (CVR) in the affected MCA territory, as demonstrated on N-isopropyl-p-[^123^I] iodoamphetamine single-photon emission computed tomography (SPECT). CVR was assessed using acetazolamide challenge. The cutoff values for resting CBF and CVR were based on the criteria established in the JET study [15].

All patients underwent superficial temporal artery to middle cerebral artery (STA-MCA) double bypass. Both the frontal and parietal branches of the STA were dissected and anastomosed in a side-to-end fashion to the supra- and infrasylvian M4 segments of the MCA, respectively [9]. Intraoperative patency was confirmed using Doppler ultrasonography [13]. Perioperative complications were evaluated during hospitalization. Postoperative MRI was performed within 3 days to confirm bypass patency. Single antiplatelet therapy was continued until the day before surgery and resumed five days after the procedure.

We analyzed the frequency and clinical characteristics of patients who developed postoperative WSIS, as well as their imaging findings, including MRI, CT angiography, digital subtraction angiography (DSA), and cerebral perfusion studies. Descriptive statistics are presented as mean ± standard deviation. Statistical analysis was performed using the χ^2^ test, and significance was defined as *p* < 0.05.

## Result

A total of 158 patients (100 males and 58 females) were included in this study. The mean age of the patients was 66.9 years. The responsible lesions of ICAD were as follows: ICA occlusion (*n* = 53), ICA stenosis (*n* = 15), M1 segment of MCA occlusion (*n* = 37), and M1 stenosis (*n* = 53). Postoperative MRI demonstrated good patency of STA-MCA bypass in all cases (100%).

Ischemic complications were observed in 3 out of 158 patients (1.9%), all of which were cases of postoperative WSIS. No other ischemic events were recorded within two weeks after surgery except one case (0.6%) suffered from intracerebral hemorrhage due to postoperative hyperperfusion.

Table 1 shows the clinical characteristics of the patients. All cases of postoperative WSIS occurred in patients with ICA stenosis (*P* < 0.05). Patients with WSIS tended to be older than those without WSIS (*P* = 0.10). No statistically significant differences were observed in sex (*P *= 1.00), preoperative CBF (*P* = 0.66), or CVR (*P* = 0.35) between the two groups. WSIS patients tended to be older than non-WSIS patients (*P* = 0.10).

**Table 1:** Clinical characteristics of the patients with and without postoperative WSIS

	WSIS (-) (*n* = 155)	WSIS (+) (*n* = 3)	*P *value
Age (years)	66.7 ± 11.8	78.0 ± 5.9	0.10
Female (*n*)	57 (36.1%)	1 (33.3%)	1.00
Affected vessel (*n*)			
* ICA stenosis*	12 (7.7%)	3 (100%)	< 0.05
* ICA occlusion*	53 (34.2%)	0	
* MCA stenosis*	53 (34.2%)	0	
* MCA occlusion*	37 (23.9%)	0	
Preoperative CBF (mL/100 g/min)	34.4 ± 5.5	33.0 ± 1.4	0.66
Preoperative CVR (%)	11.6 ± 17.5	2 ± 11.6	0.35

### Illustrative case 1 (Fig. 1)

**Fig. 1:**
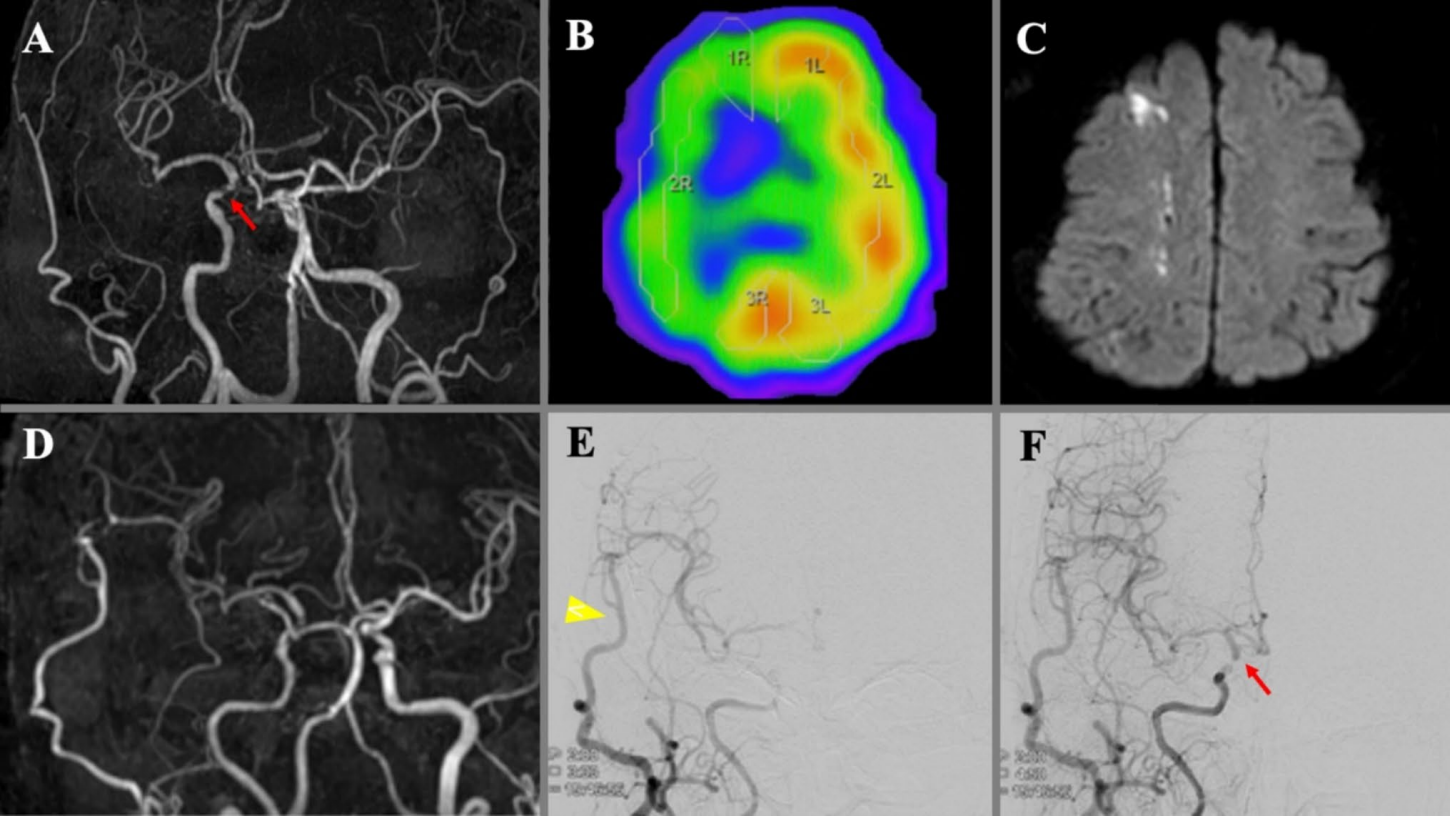
**A** Preoperative MRA showing severe stenosis of the paraophthalmic segment of the right ICA (red arrow). **B** Preoperative SPECT demonstrating markedly reduced cerebral blood flow and cerebrovascular reactivity in the affected MCA territory. **C**, **D** Diffusion-weighted MRI (**C**) on postoperative day 3 revealing a new infarction in the right watershed region, despite good bypass patency on MRA (**D**). **E**, **F** Postoperative digital subtraction angiography in the early (**E**) and late (**F**) arterial phases showing that the bypass artery (arrowhead) supplied the entire MCA territory. Anterograde flow from the right ICA terminated at the site of severe stenosis (red arrow), and perfusion of the anterior cerebral artery territory was delayed

A 71-year-old woman with recurrent transient ischemic attacks was diagnosed with severe stenosis of the right intracranial ICA. Preoperative MRA revealed approximately 85% stenosis of the paraophthalmic segment of the right ICA, as defined by the Warfarin-Aspirin Symptomatic Intracranial Disease (WASID) criteria [5]. Preoperative resting CBF and CVR in the affected MCA territory were severely decreased on preoperative SPECT. She underwent STA-MCA double anastomosis. On postoperative day 3, left hemiparesis developed, and MRI showed a new infarction in the right cortical watershed areas despite good bypass patency. Cerebral angiography revealed that the STA-MCA bypass supplied the entire MCA area, although anterograde blood flow from the right ICA terminated at the site of severe stenosis, and perfusion of the ACA area was delayed. Apparently “watershed” area had shifted towards the ACA territory. We restarted antiplatelet therapy ahead of schedule and treated her with low-molecular-weight dextran. The symptoms gradually improved, and the patient was discharged with a modified Rankin Scale (mRS) score of 2 (Fig. 1).

### Illustrative case 2 (Fig. 2)

A 79-year-old man with stenosis of the right intracranial ICA experienced a recurrent right cerebral infarction. Preoperative MRA demonstrating diminished flow signal due to approximately 90% stenosis of the petrous segment of the right ICA, as defined by the WASID criteria. He underwent STA-MCA double bypass surgery. On the third postoperative day, he developed left hemiparesis. MRI confirmed good bypass patency but revealed a new infarction in the right basal ganglia. Cerebral angiography demonstrated that the right STA–MCA bypass effectively supplied the entire peripheral MCA territory. However, anterograde flow from the right ICA was markedly diminished, and no anterograde flow was observed in the right M1 segment.

**Fig. 2:**
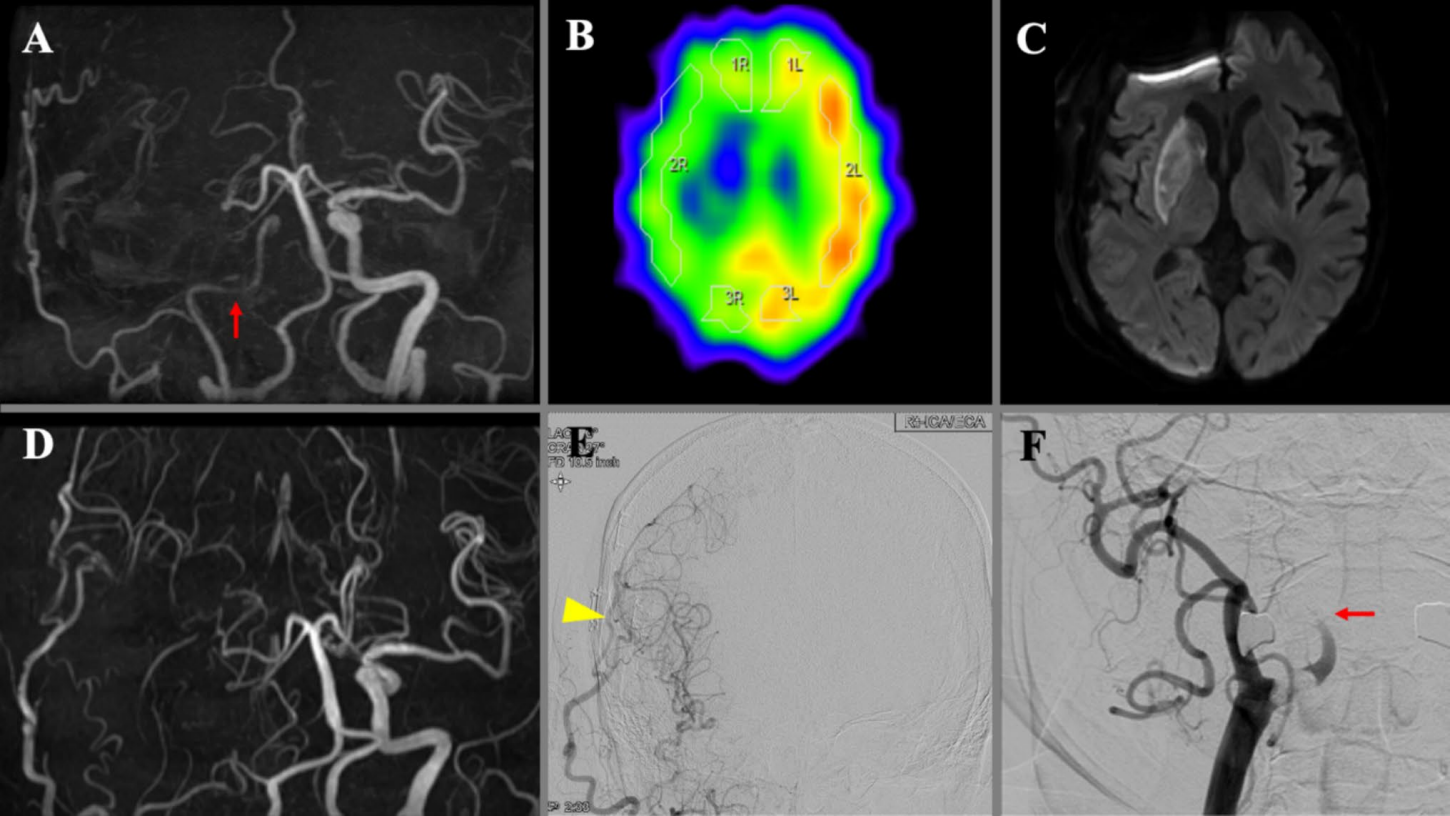
**A** Preoperative MRA shows a diminished flow signal in the right ICA due to severe stenosis at the petrous segment (red arrow). **B** Preoperative SPECT demonstrates markedly reduced cerebral blood flow and cerebrovascular reactivity in the right anterior circulation. **C**, **D **Diffusion-weighted MRI on postoperative day 3 reveals a new infarction in the right basal ganglia (**C**), despite good bypass patency confirmed by MRA (**D**). **E**, **F** Postoperative digital subtraction angiography from right intracranial (**E**) and carotid (**F**) injections shows that the STA–MCA bypass (arrowhead) supplies the entire peripheral MCA territory. Anterograde flow from the right ICA is interrupted just distal to the carotid bifurcation

### Illustrative case 3 (Fig. 3)

An 84-year-old man with a history of recurrent left cerebral infarction presented with tandem stenoses at the petrous (80%) and paraophthalmic (70%) segments of the left ICA. Preoperative SPECT revealed significantly reduced resting CBF and CVR. He underwent STA-MCA bypass surgery. Postoperatively, focal hyperperfusion was detected on SPECT imaging, requiring strict control of blood pressure. However, on the third postoperative day, he developed right hemiparesis, and MRI revealed patent bypass but new infarctions in the left corona radiata and left caudal head. Cerebral angiography demonstrated adequate blood supply through the bypass to the entire anterior circulation, although anterograde blood flow in the left ICA had disappeared.

**Fig. 3:**
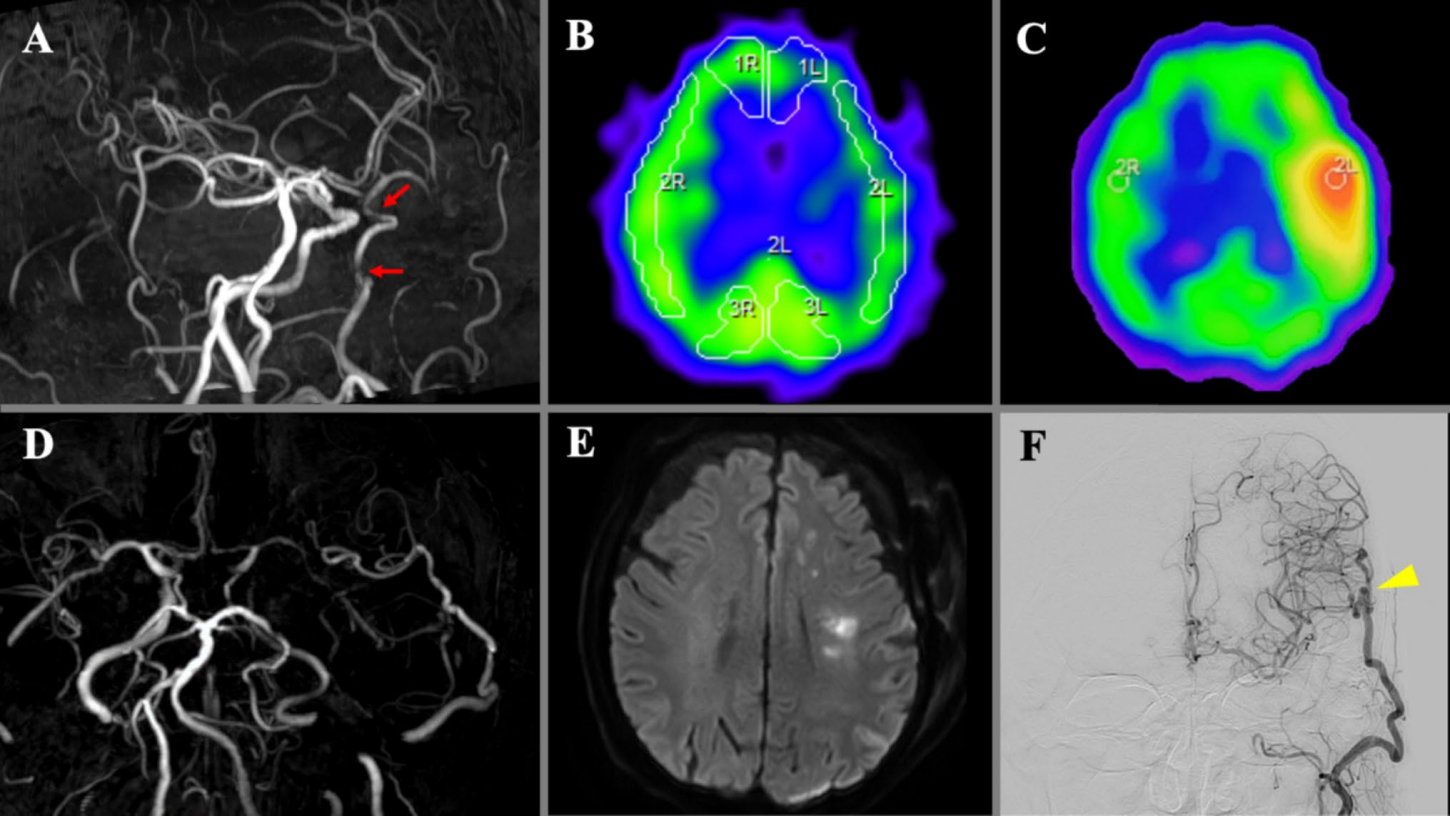
**A** Preoperative MRA shows a diminished flow signal in the left ICA due to tandem stenoses (red arrows). **B** Preoperative SPECT demonstrates markedly reduced cerebral blood flow (CBF) and cerebrovascular reactivity in the left anterior circulation. **C** Postoperative SPECT reveals focal hyperperfusion, with maximum CBF reaching 250% compared to the contralateral side. **D**, **E** On postoperative day 3, MRA (**D**) confirms good bypass patency, while diffusion-weighted imaging (**E**) shows a new infarction in the left corona radiata. **F** Postoperative left carotid angiography demonstrates adequate bypass-mediated perfusion of the entire anterior circulation, despite loss of anterograde flow in the left ICA

## Discussion

This study found that 1.9% (3/158) of all patients and 20% (3/15) of ICA stenosis patients experienced WSIS after undergoing EC-IC bypass for chronic ICAD, despite maintaining adequate bypass patency. Recent studies suggest that the ischemic complication rate following EC-IC bypass has decreased compared to the rate observed in the COSS study [2, 12, 13, 16]. However, this study highlights a higher procedural risk in patients with ICA stenosis, even in those with decreased CBF and CVR. In contrast, all ischemic complications in this cohort were attributed to WSIS in chronic ICA stenosis, suggesting that EC-IC bypass may be relatively safe in other ICAD subtypes.

These three WSIS cases shared several similarities, including severe stenosis of ICA and preserved anterograde blood flow from the ICA to MCA before surgery. However, following bypass surgery, all three cases demonstrated reduced anterograde blood flow in both the ICA and MCA. The mechanism behind WSIS seemingly involves hemodynamic watershed area shift. Retrograde blood flow from the bypass is followed by reduction of the anterograde blood flow, leading to ischemic infarctions in the newly shifted watershed areas. While not statistically significant in this study, older age may be associated with an increased risk of WSIS. Age-related impairments in cerebrovascular autoregulation, diminished collateral capacity, and increased arterial stiffness may reduce the ability to adapt to postoperative hemodynamic changes [3, 19]. These observations suggests the importance of considering ICA stenosis and older age in patient selection for STA–MCA bypass, given their potential contribution on postoperative WSIS risk.

Previous studies have reported similar phenomena only in acute phases surgery for ICAD cases. Horiuchi et al. reported two cases of severe arterial stenosis becoming occlusion after emergent EC-IC bypass surgery, probably due to hemodynamic change [8]. Kim et al. also reported four cases of hemodynamic stroke occurring 3 days after STA-MCA bypass for acute atherosclerotic occlusion [10]. It is considered that retrograde blood flow buffers the anterograde, exposing to perfusion pressure reduction risk that area. A phenomenon known as “watershed shift phenomenon (WSP)” has also been documented after EC-IC bypass for Moyamoya disease [6]. The WSP is characterized by a decrease in cerebral blood flow adjacent to the site supplied by the bypass. As a result of the bypass, blood flow may be preferentially directed to areas previously deprived of adequate blood supply, causing a shift in the watershed areas. The new watershed areas may form in different regions of the brain that were not previously at risk, potentially causing ischemic complications in these newly shifted watershed areas. In terms of hemodynamic watershed area shift, the WSP on in Moyamoya disease and WSIS in ICAD may share similar mechanisms. Although the WSP in moyamoya disease is associated with low preoperative CBF, this study did not identify any specific risk factors. Further investigation involving a larger number of cases is warranted [7, 17, 18].

Notably, all WSIS occurred on the third postoperative day, rather than immediately after the operation. This delayed onset suggests that perioperative cessation of antiplatelet therapy may also have contributed to the development of hemodynamic cerebral infarction. In this study, all patients temporarily withheld oral antiplatelet agents (primarily aspirin) from the day of surgery until postoperative day five to minimize the risk of hemorrhagic complications. The inhibitory effect of aspirin on COX-1 lasts for approximately 7–10 days, corresponding to the lifespan of platelets, but bleeding time reportedly returns to normal within 2 days after drug withdrawal as new platelets are produced [1]. Thus, the development of WSIS on postoperative day three may reflect attenuation of the antiplatelet effect. Other potential factors affecting cerebral perfusion during the perioperative period include strict control of the arterial blood pressure to prevent hyperperfusion syndrome after EC-IC bypass surgery [9, 14]. Although no sustained hypotensive episodes were documented in patients with WSIS, transient hypotension may still contribute to the risk of hemodynamic ischemic stroke. Therefore, for future management, normotensive postoperative care and early resumption of antiplatelet therapy should be considered to prevent WSIS after EC-IC bypass.

Lastly, since all patients in this study underwent double-barrel bypass procedures, it remains unclear whether the incidence of WSIS would differ in cases of single-barrel bypass. Double-barrel bypass is generally considered to perfuse a broader cortical territory, which might theoretically induce a greater watershed shift [4]. Additionally, intraoperative STA flow may also influence the postoperative flow dynamics. Future studies should incorporate single-barrel cases and intraoperative flow measurements to clarify how bypass configuration and flow influence WSIS risk.

## Conclusion

The present study evaluated the characteristics of WSIS following STA-MCA bypass in patients with chronic ICAD. The findings indicate that cases of ICA stenosis with preserved anterograde blood flow of MCA are at risk, and hemodynamic stroke may manifest within the few days following STA-MCA bypass surgery.

## Data Availability

No datasets were generated or analysed during the current study.
